# Syringocystadenocarcinoma papilliferum at an uncommon location in an immunocompromised patient: A case report and literature review

**DOI:** 10.1016/j.jdcr.2025.08.012

**Published:** 2025-08-20

**Authors:** Iswarya Shree Nee, Peddireddy Sreekar

**Affiliations:** Department of Dermatology, Sri Venkateswara Medical College, Tirupati, Andhra Pradesh, India

**Keywords:** adnexal tumor, apocrine, apocrine carcinoma, axilla, case report, glandular, histopathology, HIV, immunocompromised, literature review, Mohs, nuclear atypia, papillary, rare skin cancer, SCAP, syringocystadeno papilliferum, syringocystadenocarcinoma papilliferum, uncommon, wide excision

## Introduction

Syringocystadenocarcinoma papilliferum (SCACP) is a rare malignant adnexal tumor of apocrine origin, most commonly arising in the head and neck regions.^1^ It typically develops from its benign precursor, syringocystadenoma papilliferum (SCAP), which often presents as a slow-growing, verrucous lesion for many years. When congenital, SCAP is frequently associated with nevus sebaceous of Jadassohn.^2^

Although uncommon, SCACP can exhibit aggressive behavior, with documented cases of both locoregional and distant metastases. Surgical excision remains the primary treatment approach.^3^

In this report, we present a rare case of SCACP occurring in the axilla of an immunocompromised patient, along with a review of relevant literature.

## Case presentation

A 74-year-old female with a known history of HIV infection on antiretroviral therapy for the past 10 years presented with a progressively enlarging ulcerative lesion in the left axilla, present for 5 months. The lesion began as a bean-sized papule and gradually increased in size over a short period. Later, it became painful and ulcerated.

**Fig 1:**
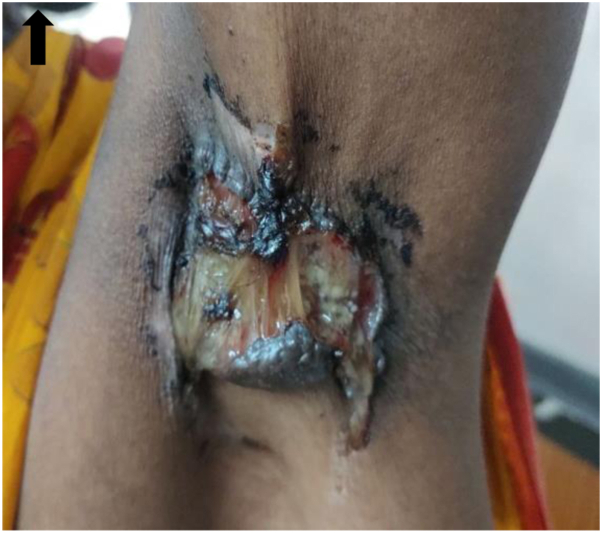
Clinical image of ulcerated lesion in the left axilla. An upward arrow indicates the top edge. The image shows the clinical appearance of the lesion, revealing irregular margins, mucoid discharge, and hemorrhage along the indurated edges.

Cutaneous examination revealed a single irregularly shaped ulcer measuring 6 × 4 × 2 cm, with a necrotic slough-covered base, erythematous margins, and surrounding induration. The surface showed areas of hemorrhage and mucoid discharge (Fig 1).

Regional lymph nodes were not palpable, and the physical examination was unremarkable apart from the presence of the ulcer.

**Fig 2:**
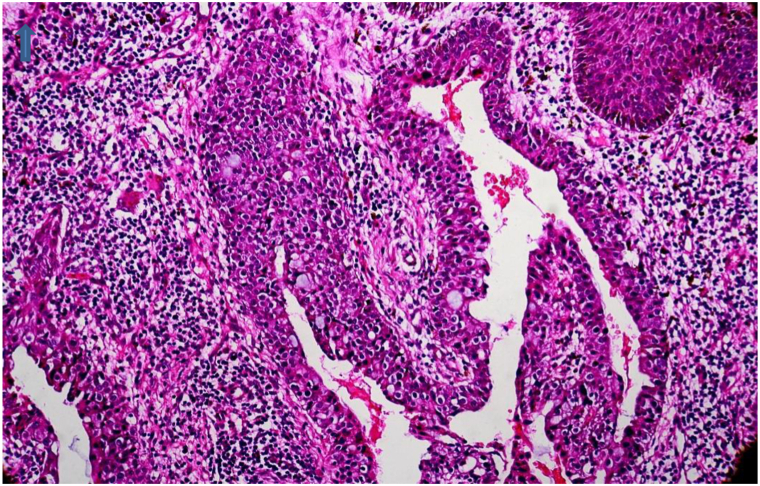
Papillary and glandular proliferation in the dermis (low-power view). An upward arrow marks the top edge. The image illustrates a papillary structure with an irregular lumen, cytological atypia, and an inflammatory infiltrate (hematoxylin and eosin stain).

**Fig 3:**
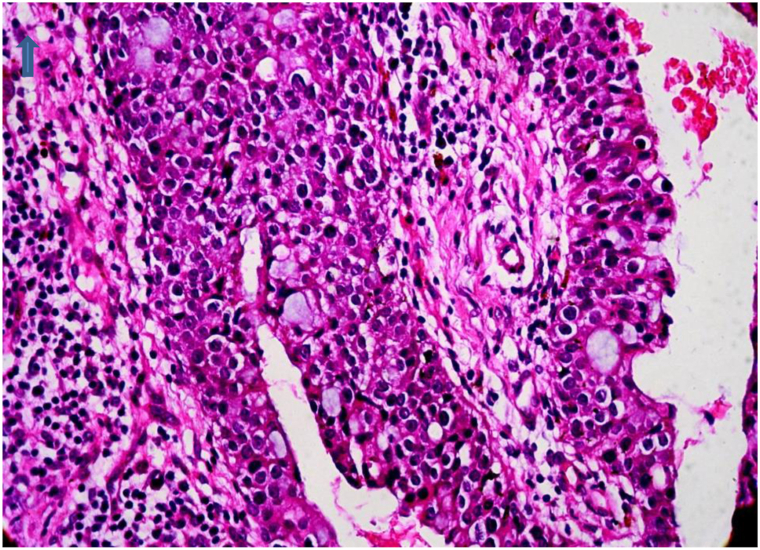
Atypical glandular structure with papillary projections (high-power view). An upward arrow designates the top edge. The figure demonstrates marked nuclear pleomorphism, high mitotic activity, and a dense plasmacytic infiltrate (hematoxylin and eosin stain).

Histopathological examination revealed a papillomatous growth in the upper and mid-dermis consisting of villi with double-layered epithelium composed of cuboidal and columnar cells with pleomorphic nuclei. The papillary processes were supported by a fibrovascular core containing lymphocytes and plasma cells. No obvious metastatic nodules or vascular invasion were seen, and the deep dermis and fat were uninvolved. The findings were suggestive of SCACP (Figs 2 and 3).

Laboratory investigations revealed normal baseline parameters. Imaging studies, including computed tomography of the chest and abdomen as well as whole-body fluorodeoxyglucose positron emission tomography, were conducted to assess for locoregional or distant metastasis. No metastasis was detected beyond the primary axillary tumor. Based on these findings, a diagnosis of SCACP was rendered.

The patient underwent wide excisional surgery, and histopathological analysis confirmed tumor-free margins.

## Discussion

A systematic review of the literature on SCACP was conducted following the Preferred Reporting Items for Systematic Reviews and Meta-Analyses guidelines.^4^ We reviewed all available case reports and series published in English literature to compile relevant data on this rare malignancy.

To date, only 81 cases of SCACP have been reported in English literature, making our case the 82nd documented instance. Additionally, there is only 1 previously reported case of SCACP in an immunocompromised patient, and our case represents the second.^5^

The most common site of involvement is the head and neck, observed in 44 cases, whereas axillary involvement is rare. Including our case, only 5 cases of axillary SCACP have been documented (Table I).^1^, ^2^, ^3^^,^^5^^,^^6^

**Table I:** Tumor sites and their respective case numbers with percentages among the 82 reported cases

Site	Number of cases
Head	41 (50%)
Neck	3 (3.6%)
Chest	7 (8.5%)
Back	3 (3.6%)
Abdomen	2 (2.4%)
Axilla	5 (6.1%)
Extremity	12 (14.6%)
Genitalia	3 (3.6%)
Perianal	6 (7.3%)

Our analysis indicates that SCACP predominantly affects the elderly population, with a median age of 65.8 years (range 15-96). Only 10 cases have been documented in individuals under 40 years of age. There is a slight male predominance, with a male-to-female ratio of 1.3:1.

Clinically, the lesion often presents as a slow-growing nodule persisting for decades, followed by a sudden increase in size, ulceration, crusting, and mild pain. Lesions may present as nodules, plaques, ulcers, or tumors.^7^

SCACP is frequently associated with benign adnexal tumors, including SCAP, trichoblastoma, and trichilemmoma.

SCACP is characterized histopathologically by a cystic and papillary growth within the epidermis and dermis, with deep epidermal invaginations containing papillary projections. These are lined by a dual-layered epithelium, consisting of an inner columnar layer with oval nuclei, eosinophilic cytoplasm, and decapitation secretion, and an outer cuboidal layer with scant cytoplasm and small, oval nuclei.^8^^,^^9^ SCACP differs from SCAP by its asymmetric tumor architecture and ill-defined boundaries.^10^ Additionally, neoplastic cells exhibit crowded nuclei, varying degrees of nuclear atypia, and a high mitotic rate.^7^^,^^11^, ^12^, ^13^, ^14^, ^15^

The tumor stains positive for cytokeratin 5/6, p63, and D2-40 on immunohistochemical analysis; however, these markers are not entirely specific.^7^^,^^12^^,^^16^^,^^17^

Clinically, ulcerative lesions in the axilla should be carefully differentiated from other conditions, including squamous cell carcinoma, basal cell carcinoma, cutaneous tuberculosis (such as scrofuloderma), deep fungal infections, and necrotizing soft tissue infections.^18^^,^^19^

Histologically, the differential diagnosis includes hidradenoma papilliferum, extramammary Paget’s disease (including anaplastic variants), and cutaneous metastases from breast, gastrointestinal, or thyroid carcinoma. SCAP with atypia can resemble SCACP, especially when there is architectural complexity and nuclear atypia but without malignant features. Anaplastic Paget’s disease may also mimic SCACP due to glandular features and pleomorphism but usually shows diffuse intraepidermal spread and lacks the papillary structures and stromal inflammation typical of SCACP.^13^^,^^20^, ^21^, ^22^

Wide local excision is the standard treatment for SCACP. Among 82 reported cases, treatment approaches included excision, Mohs surgery, radiotherapy, chemotherapy, exenteration, and combinations thereof (Table II). Excision was used in 57 cases (69%), with some receiving additional lymphadenectomy (4 cases), chemotherapy (4 cases), or radiotherapy (1 case). Mohs surgery was performed in 3 cases (4%), while treatment details were unavailable for 18 cases.

**Table II:** Treatment approaches for SCACP and their corresponding case numbers among the 82 reported cases

Treatment modalities	Number of cases
Excision	48
Excision plus lymphadenectomy	4
Excision plus chemotherapy	4
Excision plus radiotherapy	1
Mohs	4
Radiotherapy	1
Chemotherapy	2
Not available	18

*SCACP*, Syringocystadenocarcinoma papilliferum.

Follow-up outcomes showed that 39 had no recurrence or metastasis, 3 experienced local recurrence, 10 developed metastasis, 3 deaths occurred due to other causes, and 27 had outcomes that were not available or not mentioned. Mohs surgery showed no documented recurrences or metastasis, indicating potentially superior margin control. Wide excision was mostly effective but occasionally recurred. Nonspecified excisions had mixed results, including instances of local recurrence and metastasis.^1^, ^2^, ^3^^,^^5^, ^6^, ^7^^,^^11^^,^^12^^,^^16^^,^^20^, ^21^, ^22^, ^23^, ^24^, ^25^, ^26^, ^27^, ^28^, ^29^, ^30^, ^31^, ^32^, ^33^, ^34^, ^35^, ^36^, ^37^, ^38^, ^39^, ^40^, ^41^, ^42^, ^43^, ^44^, ^45^, ^46^, ^47^, ^48^, ^49^, ^50^, ^51^, ^52^, ^53^, ^54^, ^55^, ^56^, ^57^, ^58^, ^59^, ^60^, ^61^

This rare axillary SCAP case underwent malignant transformation into SCACP. The absence of any preceding or long-standing lesion clinically suggests a de novo presentation of SCAP, which subsequently transformed malignantly.

Although SCACP in immunocompromised patients is rare, most reported cases occur in immunocompetent individuals, suggesting the association may be coincidental rather than causal. Given the rarity of this malignancy, our findings underscore the importance of considering SCACP in the differential diagnosis of atypical ulcerative lesions in the axilla, ensuring timely diagnosis and appropriate management.

## Conflicts of interest

None disclosed.
